# DNA Virus Detection in Olfactory Neuroblastomas Using Targeted Enrichment NGS

**DOI:** 10.1111/neup.70055

**Published:** 2026-03-24

**Authors:** Maria K. Jauhiainen, Outi I. Mielonen, Aaro Haapaniemi, Maria Söderlund‐Venermo, Jaana Hagström, Maria F. Perdomo, Saku T. Sinkkonen, Antti A. Mäkitie

**Affiliations:** ^1^ Department of Otorhinolaryngology—Head and Neck Surgery Helsinki University Hospital and University of Helsinki Helsinki Finland; ^2^ Department of Virology University of Helsinki and Helsinki University Hospital Helsinki Finland; ^3^ Research Program in Systems Oncology, Faculty of Medicine University of Helsinki Helsinki Finland; ^4^ Department of Pathology Helsinki University Hospital Helsinki Finland; ^5^ Department of Oral Pathology and Radiology University of Turku Turku Finland; ^6^ Division of Ear, Nose and Throat Diseases, Department of Clinical Sciences, Intervention and Technology Karolinska Institute and Karolinska University Hospital Stockholm Sweden

**Keywords:** DNA viruses, human papillomavirus viruses, olfactory esthesioneuroblastoma, pathology, sequence analysis

## Abstract

A variety of malignancies can be found in the sinonasal tract including the nasal cavity, paranasal sinuses, and skull base. Many of these are attributed to viruses, for example, carcinomas with the presence of transcriptionally active high‐risk human papillomavirus, as well as Epstein–Barr virus associated malignant lymphoepithelial carcinoma and haematolymphoid neoplasias. Olfactory neuroblastoma (ONB) is a rare malignant tumor of the olfactory fossa with an unknown etiology. We present the first comprehensive virus study of ONB. By targeted next‐generation sequencing for 41 DNA viruses, we investigated the presence of papillomaviruses, herpesviruses, polyomaviruses, and parvoviruses, among others, in 12 ONBs. In addition, herpesvirus presence was evaluated by qPCR. Low‐risk HPV6 was detected in one sample. Human endogenous retroviruses were positive in all samples, supporting successful library preparation and sequencing, considered as an internal quality control. No other virus findings were observed. The present broad‐scale virus study did not reveal abundant presence of viral DNA in ONB suggesting a limited viral role in its etiology. Nevertheless, our cohort exhibited a finding of low‐risk HPV, which has been associated with increased risk of cancer progression of inverted sinonasal papilloma in the same anatomic site advocating for further research.

**Trial Registration:** §31/07.03.2019

**Abbreviations**
BLAST: basic local alignment search tool
CDKN2A: cyclin‐dependent kinase inhibitor 2A
CMV: cytomegalovirus
DNA: deoxyribonucleic acid
EBV: Epstein–Barr virus
EGFR: epidermal growth factor receptor
FFPE: formalin‐fixed paraffin‐embedded
HERV: human endogenous retrovirus
HHV: human herpesvirus
HNSCC: head and neck squamous cell carcinoma
HPV: human papillomavirus
KSHV: kaposis sarcoma herpesvirus
NGS: next‐generation sequencing
ONB: olfactory neuroblastoma
PIK3CA: phosphatidylinositol‐4,5‐bisphosphate 3‐kinase, catalytic subunit alpha
qPCR: quantitative polymerase chain reaction
Rnase P: ribonuclease P
TP53: tumor protein 53

## 1 Introduction

Infectious agents contribute to approximately 15% of cancers worldwide [1, 2]. Human papillomavirus (HPV) causes cervical and oropharyngeal carcinomas among others [3, 4], Epstein–Barr virus (EBV) is the etiologic factor for nasopharyngeal carcinoma, and oncogenic viruses have been identified within several other virus families as well. Modern approaches have enabled rapid progression in virus research, and new viruses have been discovered within the recent decades, exemplified by polyomaviruses and protoparvoviruses [5, 6]. Whereas the oncogenic pathways for viruses such as HPV and EBV are studied intensively, the roles of many others are only starting to unveil. Moreover, it has been recognized that indirect actions of viruses in carcinogenesis are multifaceted, and even without a direct oncogenic effect, viruses may influence the tumor microenvironment and contribute to tumor formation [7, 8].

Olfactory neuroblastoma (esthesioneuroblastoma, ONB) is a rare malignant tumor of the olfactory fossa, arising from the olfactory epithelium. The annual incidence of ONB is approximately 0.4 per million inhabitants [9], affecting most commonly middle‐aged persons, but it can be encountered even in children. A multimodal therapeutic strategy including surgical treatment, radiotherapy, and chemotherapy is recommended [10] but recurrences and metastases are not uncommon.

The etiology of ONB is unknown [11]. Exposure to radiation or radiotherapy have been suggested as possible agents [12, 13]. Alterations in genes, such as *TP53, PIK3CA, CDKN2A*, and *EGFR*, among others, have been reported in the majority of ONBs, with *TP53* being the most prevalent mutated gene [14, 15, 16]. Overall, *TP53* is commonly mutated in various cancers and encountered frequently among head and neck squamous cell carcinomas (HNSCCs). The degradation of tumor suppressor protein p53 is a well‐characterized oncogenic activity of HPV16 type oncoprotein E6 within HPV‐positive squamous cell carcinomas [17]. Overall, both pathways usually lead to a similar result, possibly compromising the function of the tumor suppressor. The *DCKN2A* gene, in turn, codes for proteins such as p16, where the overexpression reflects the presence of high‐risk HPV [18]. PIK3CA mutations, in turn, have been linked to various cancers, including EBV‐associated gastric carcinoma with overexpression related to a favorable impact on survival [19, 20].

Virus presence in human ONB has been studied regarding only one virus. The studies were performed in 1999 and 2007, with cohort sizes of 16 and 9 ONBs, which analyzed the presence of EBV with no findings [21, 22]. Within animal studies, activation of retrovirus was observed in transgenic mice with ONB, and type C retrovirus was observed in a case series of cats with spontaneous ONB, serologically positive for feline leukemia virus [23, 24]. However, these series have been small, and no causal relationship has been established.

The unknown etiology of ONB, the massive exposure to viruses due to the anatomical location in the nasal cavity, and the unknown role of many novel viruses justify this study. Hence, we explored the presence of a wide variety of DNA viruses in ONB by targeted next‐generation sequencing (NGS) and qPCR.

## 2 Materials and Methods

### 2.1 Ethics

The Helsinki University Hospital Ethical Committee approved the study design (§31/07.03.2019) and a research permission was granted (HUS/332/2019). The Declaration of Helsinki guidelines have been followed within this study.

### 2.2 Patients and Clinical Specimens

The cohort comprised 12 consecutive patients diagnosed during 1985–2019 and with tumor samples available at the Helsinki Biobank (permission no §73/15.05.2019, HUS/118/2019). By virtue of the Helsinki Biobank Act, informed consent is implicit for these individuals. The patients were treated at the Departments of Otorhinolaryngology—Head and Neck Surgery and Oncology, Helsinki University Hospital, Helsinki, Finland. We recorded the patient‐ and tumor‐related parameters from hospital charts.

Altogether, we analyzed 15 formalin‐fixed, paraffin‐embedded (FFPE) samples from 12 patients (labeled as ONB01‐ONB12). Patients 8, 10, and 12 had 2 samples each (taken from different tumor blocks). Tumor‐rich regions of FFPE samples were collected in a PCR‐sterile manner from paraffin tissue blocks as 2‐mm punch biopsies in 1.5 mL microcentrifuge tubes.

### 2.3 DNA Extraction

We extracted DNA from the FFPE biopsy samples with QIAamp DNA FFPE Tissue Kit (Qiagen, Heiden, Germany), according to the manufacturer's protocol, with slight modifications as described previously [25]. We evaluated the DNA yields and human cell quantity by comparing viral loads with that of the human reference single‐copy gene *RNase P* by qPCR [26]. Molecular biology grade water was included as negative controls. Strict precautions were implemented during sample handling and processing, including the use of disposable consumables and filter tips, dedicated hoods for nucleic acid work, and the inclusion of non‐template controls at all analytical steps.

### 2.4 Virus DNA Detection by NGS

For virus detection we applied custom‐targeted enrichment by NGS (Table 1) [27, 28]. In brief, the DNA was fragmented, and we prepared the libraries with KAPA HyperPlus kit (Roche) using Unique Dual Index Adapters. Molecular biology grade water was included as negative controls. Targeted enrichment was performed using a custom panel of biotinylated RNA‐probes covering the full viral genomes (Arbor Biosciences). Two rounds of hybridization for all samples were done individually. We amplified the libraries 3 × 13‐25 cycles during library preparation and the enriched libraries were quantified with the KAPA Library Quantification Kit (Roche) using Stratagene 3005P qPCR System (Agilent) and pooled for sequencing on NovaSeq 6000 (S1, PE151 kit; Illumina).

**TABLE 1:** Targeted viruses investigated by NGS.

Papillomaviruses	Herpesviruses	Parvoviruses	Polyoma‐viruses
HPV2	Herpes simplex 1	Human parvovirus B19	BKPyV
HPV6	Herpes simplex 2	Human bocavirus 1	JCPyV
HPV11	Varicella Zoster	Human bocavirus 2	KIPyV
HPV16	Epstein Barr virus	Human bocavirus 3	WUPyV
HPV18	Cytomegalovirus	Human bocavirus 4	MCPyV
HPV31	Human herpesvirus 6A	Cutavirus	HPyV 6
HPV45	Human herpesvirus 6B		HPyV 7
	Human herpesvirus 7		TSPyV
	Kaposi sarcoma virus		HPyV 9
			MWPyV
**Anelloviruses**	**Hepadnaviruses**	**Poxviruses**	STLPyV
Torque teno virus 1	Hepatitis B virus	Variola major virus	HPyV 12
Torque teno virus 10		Variola minor virus	NJPyV
Torque teno virus 13			Simian virus 40

*Note:* Herpesviruses were analyzed both by NGS and qPCR.

Abbreviations: HPV, human papillomavirus; PyV, polyomavirus.

### 2.5 Sequence Data Analysis

We reconstructed viral genomes from the sequencing data with TRACESPipe, a fully automatic tool providing reconstructed viral genomes, the consensus sequences, breadth and depth coverage, and the associated profiles, among other information and quality controls [29]. In brief, we utilized FALCON‐meta to find the highest similar reference from the NCBI viral database [30]. For aligning the reads, we used the Burrows–Wheeler alignment tool [31] and reconstructed the consensus sequences (SAMtools, BCFtools) [32]. The coverage profiles were created with BEDtools [33]. Individual sequences, when in low coverage (< 15%), were manually inspected and confirmed by BLAST (Basic Local Alignment Search Tool; NIH National Library of Medicine, Rockville, Bethesda, MD, USA).

### 2.6 Virus DNA Detection by qPCR

We quantified by qPCR the cell copy numbers (RNAse P) and herpesviruses due to their ubiquitous nature, and possibly neurotropic behavior (Table 1). All human herpesviruses were included in multiplex assays: herpes simplex‐1 and ‐2, varicella zoster, EBV, cytomegalovirus (CMV), human herpesvirus (HHV)‐6A, ‐6B, and ‐7, and Kaposis sarcoma herpesvirus (KSHV). All virus qPCR reactions occurred in TaqPath ProAmp Multiplex Master Mix (Thermo Fisher Scientific). We performed all qPCR assays with AriaMx Realtime PCR System (Agilent Technologies, Santa Clara, CA, USA) [34]. Molecular biology grade water was included in all PCR runs as a non‐template control. Ten‐fold diluted plasmids (10^1^–10^6^), containing each viral target amplicon, served as qPCR standards and as positive controls. The human reference gene *RNa*se P served as control for DNA yield and human cell quantity. Reaction mix preparation, DNA extraction, plasmid controls, and amplification procedures were conducted in physically separated rooms to minimize the risk of contamination.

## 3 Results

### 3.1 Patient and Tumor Characteristics

**FIGURE 1:**
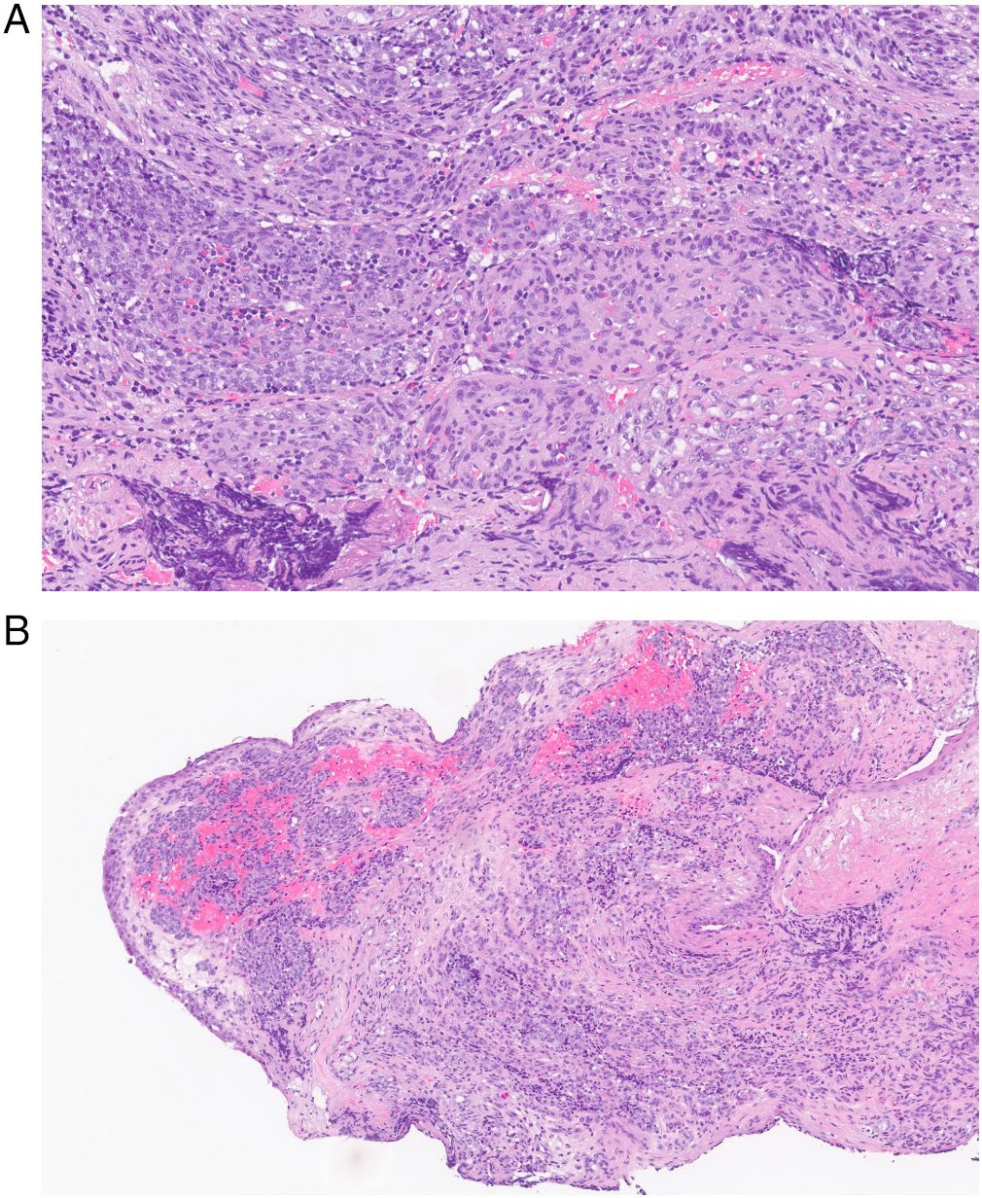
Low grade olfactory neuroblastoma (Hyams grade 1–2) with low mitotic activity and without tumor necrosis. (A) High‐power field. (B) Low‐power field.

The patient characteristics are described in Table 2. The mean age at diagnosis was 56.5 years. Most tumors represented Hyams low‐grade malignancy (Table 2 and Figure 1) [35].

**TABLE 2:** Patient characteristics.

Patient ID	Sex	Age	Comorbidities	Hyams grade
ONB1	M	64	N/A	Low grade (gr 1–2)
ONB2	M	58	N/A	Low grade (gr 2)
ONB3	F	75	N/A	Low grade
ONB4	M	46	N/A	Low grade
ONB5	M	74	Hypertension, asthma, stroke, colon carcinoma	N/A
ONB6	M	47	N/A	Low grade (gr 2)
ONB7	F	23	N/A	N/A
ONB8	M	54	DM2, hypertension	Low grade
ONB9	F	21	Healthy (smoker)	High grade (gr 3)
ONB10	M	65	Healthy	Low grade (gr 2)
ONB11	F	81	DM2, hypertension	Low grade (gr 2)
ONB12	F	64	DM1, Addison's disease, rheumatic arthritis	Low grade (gr 2)

Abbreviations: DM1, diabetes mellitus type 1; DM2, diabetes mellitus type 2; F, female; gr, grade; M, male; N/A, not available.

### 3.2 Presence of Virus by NGS

All tumor samples were analyzed by NGS. Human endogenous retrovirus (HERV) served as an internal control, and all samples were HERV positive. Sample ONB1 was positive for HPV6 with five manually confirmed reads mapping exclusively to this virus by BLAST. All other samples were negative for all the 41 tested viruses.

### 3.3 Presence of Virus by qPCR

*RNase P‐*qPCR results varied between 10^2^–10^4^ copies/ml among all samples with a mean of 5.6 × 10^3^ (Table 3). All herpesviruses tested negative in the analyses. For patients ONB 8, 10, and 12, who had two distinct samples taken, the *RNase P* and cell quantities are shown as mean values, and all samples were HERV positive but negative for other viruses.

**TABLE 3:** Cell quantity and virus findings by NGS and qPCR.

Patient ID	Cell quantity (cells/μL)	NGS HERV	NGS	Herpes‐viruses (qPCR)
ONB1	7.0 × 10^3^	HERV	HPV6	neg
ONB2	8.5 × 10^2^	HERV	neg	neg
ONB3	6 × 10^3^	HERV	neg	neg
ONB4	2.8 × 10^3^	HERV	neg	neg
ONB5	1.4 × 10^3^	HERV	neg	neg
ONB6	2.7 × 10^2^	HERV	neg	neg
ONB7	5.0 × 10^2^	HERV	neg	neg
ONB8	2.9 × 10^3^	HERV	neg	neg
ONB9	2.6 × 10^3^	HERV	neg	neg
ONB10	4.8 × 10^3^	HERV	neg	neg
ONB11	4.1 × 10^3^	HERV	neg	neg
ONB12	1.9 × 10^3^	HERV	neg	neg

Abbreviations: HERV, human endogenous retrovirus; HPV, human papillomavirus.

## 4 Discussion

Some of the malignant tumors of the sinonasal tract harbor viruses as etiological factors associated with carcinoma development. These include non‐keratinizing squamous cell carcinoma often positive for high‐risk HPV, HPV‐related multiphenotypic sinonasal carcinoma defined by the presence of transcriptionally active high‐risk HPV, as well as sinonasal lymphoepithelial carcinoma mainly associated with EBV. We analyzed the presence of papilloma‐, herpes‐, polyoma‐, and parvoviruses, among other viruses, in ONB. Both sequencing and qPCR were utilized, with very few positive findings altogether. HERVs, human endogenous retroviral sequences, were detected in all samples, indicating successful library preparation and sequencing.

ONB is a genetically heterogenous tumor derived from immature or progenitor olfactory epithelial cells [36]. Genetic mutations, present in other tumor entities as well, have been reported [14, 15, 16], but due to ONB's rarity, clinical research studies examining tumorigenesis' genetic and biochemical processes have been limited (Demir [37]). Accordingly, the unknown etiology of ONB may be multifaceted reflecting its heterogenous phenotype.

Very few virus investigations have been conducted regarding ONB, and HPVs, despite their importance in other tumors, have not previously been analyzed in this context. HPV‐driven carcinomas of the upper airways are traditionally associated with the oropharynx. However, HPV positivity has also been detected in other tumors of the nasal cavity and nasopharynx, with prevalence varying geographically and across anatomical subsites [38].

Various HPV types have high‐risk or low‐risk oncogenic potential in humans. In our study, HPV6 was present in one ONB of a 64‐year‐old male with a low‐grade malignancy. Low‐risk HPV6 is a major cause of benign tumors such as anogenital warts and laryngeal papillomas. Although HPV6 has been detected in malignant tumors of the head and neck as the most prevalent finding after high‐risk HPV16 and 18, there is no established causal association with malignant transformation [38]. In anatomically closely related tumors of the sinonasal tract, such as in sinonasal inverted papillomas (SNIP), HPV6 is the most prevalent HPV subtype [39]. Recent studies indicate that low‐risk HPV infection may increase the risk of malignant progression, in contrast to non‐keratinizing squamous cell carcinomas conventionally positive for high‐risk HPV [40, 41].

In the sinonasal tract, EBV has been strongly associated with sinonasal lymphoepithelial carcinoma [42, 43]. Within our cohort of ONBs, herpesvirus analyses were investigated both by NGS and qPCR to evaluate the presence of EBV and other herpesviruses, including the neurotropic HSV‐1, ‐2, and varicella zoster virus. The negative findings of EBV are consistent with previous studies [21, 22]. Even human herpesviruses‐6 and ‐7 were negative, although they have been rather frequently detected in tumors of the sinonasal area in our other studies [44, 45].

The relatively small size of our cohort, due to the rarity of ONB, and the included tumors representing mainly low‐grade tumors without the information of molecular subtype, form a limiting factor of the present study. In addition, the patients represent a geographically limited population (Finland). However, possible DNA damage induced by the fixation may also be a limitation, albeit our internal controls (RNAse P and HERVs) provide internal validation. Of note, we have previously demonstrated the efficient use of FFPE and validated the methods used in this study in formalin‐fixed samples, showing good sensitivity and reproducibility [46]. All samples tested positive for HERV, indicating that the DNA in the tumor samples is still in good condition and highlighting the potential utility of NGS with targeted enrichment for viral genome detection, for example in diagnostic applications. However, viral mRNA studies for virus activity and in situ studies for target cell typing could provide more pathobiological insights, however, mRNA is challenging to be amplified from such FFPE samples due to RNA fragmentation. The viral load was also too low for ISH. In addition, our study lacked healthy control tissue, and the results can therefore only be compared to those of earlier tissue studies of the same anatomical area [21, 38, 42, 43, 44, 45, 47, 48]. Therefore, virus screening from the fresh‐frozen specimens of the normal olfactory epithelium and fresh ONB tumors of all histological subtypes could enhance sensitivity for, for example, mRNA studies and provide valuable insights.

No previous extensive virus analyses of ONB have been performed, emphasizing the importance of the present study and its results. Although a viral background is a relevant factor in multiple malignancies, the DNA‐virus findings in our study were scarce with a few reads only. This does not rule out possible viral involvement considering, for example, potential early oncogenic hit‐and‐run events [7], and potential RNA virus involvement, which warrant further investigations. Our cohort presented the first finding of low‐risk HPV in ONB. Based on the current knowledge, low‐risk HPVs may increase the malignant progression of sinonasal inverted papillomas in the same anatomical location.

## Funding

This work was supported by Finska Läkaresällskapet, Finnish Medical Foundation (7805), state funding for Helsinki University Hospital Research (TYH2024327), Sigrid Jusélius Foundation, and Kirsti and Tor Johansson Heart and Cancer Foundation.

## Ethics Statement

The Helsinki University Hospital Ethical Committee approved the study design (§31/07.03.2019) and a research permission was granted (HUS/332/2019). The Declaration of Helsinki guidelines have been followed within this study.

## Consent

The management of samples was done in accordance with the Biobank act (688/2012).

## Conflicts of Interest

The authors declare no conflicts of interest.

## Data Availability

The data that support the findings of this study are available on request from the corresponding author. The data are not publicly available due to privacy or ethical restrictions.
